# To target cellular senescence in diabetic kidney disease: the known and the unknown

**DOI:** 10.1042/CS20240717

**Published:** 2024-08-14

**Authors:** Yuehan Wei, Shan Mou, Qing Yang, Fang Liu, Mark E. Cooper, Zhonglin Chai

**Affiliations:** 1 Department of Diabetes, School of Translational Medicine, Monash University, Melbourne, Australia; 2 Department of Nephrology, Molecular Cell Laboratory for Kidney Disease, Shanghai Peritoneal Dialysis Research Center, Ren Ji Hospital, Uremia Diagnosis and Treatment Center, Shanghai Jiao Tong University School of Medicine, Shanghai, China; 3 Department of Nephrology, Laboratory of Diabetic Kidney Disease, Kidney Research Institute, West China Hospital, Sichuan University, Chengdu, China

**Keywords:** Cellular Senescence, diabetic nephropathy, inlfammation, renal fibrosis

## Abstract

Cellular senescence represents a condition of irreversible cell cycle arrest, characterized by heightened senescence-associated beta-galactosidase (SA-β-Gal) activity, senescence-associated secretory phenotype (SASP), and activation of the DNA damage response (DDR). Diabetic kidney disease (DKD) is a significant contributor to end-stage renal disease (ESRD) globally, with ongoing unmet needs in terms of current treatments. The role of senescence in the pathogenesis of DKD has attracted substantial attention with evidence of premature senescence in this condition. The process of cellular senescence in DKD appears to be associated with mitochondrial redox pathways, autophagy, and endoplasmic reticulum (ER) stress. Increasing accumulation of senescent cells in the diabetic kidney not only leads to an impaired capacity for repair of renal injury, but also the secretion of pro-inflammatory and profibrotic cytokines and growth factors causing inflammation and fibrosis. Current treatments for diabetes exhibit varying degrees of renoprotection, potentially via mitigation of senescence in the diabetic kidney. Targeting senescent cell clearance through pharmaceutical interventions could emerge as a promising strategy for preventing and treating DKD. In this paper, we review the current understanding of senescence in DKD and summarize the possible therapeutic interventions relevant to senescence in this field.

## Introduction

Cellular senescence is a physiological and natural process in organic systems. The word ‘senescence’ originates from the Latin word ‘senex’ which means ‘old man’ or ‘old age.’ In 1891, Minot used the word to describe the passage of youth to old age in the biological context [1] and in the 1960s, Hayflick and Moorhead described the degeneration and passage limitation of human fibroblasts in culture, which was first explained by ‘senescence’ [2]. However, at that time, the concept of senescence was unclear both at the cellular level and at the level of the organism.

The understanding of senescence has significantly advanced over the last few decades. Initially, the phenomenon of cellular senescence referred to events in cultured cells where cell replication was attenuated with age. The current definition refers to a permanent cell growth arrest state in response to distinct stresses, such as oncogene activation, DNA damage and oxidative stress [3]. Since senescence is a phenotype, resulting from multiple effector mechanisms, the identification of senescence is based on the presence of a combination of multiple biomarkers, such as senescence-associated β-galactosidase (SA-β-Gal), cell cycle arrest, senescence-associated secretory phenotype (SASP), DNA damage response (DDR), abnormal expansion and flattening in cell morphology [4,5].

Diabetic kidney disease (DKD) is the most common case of chronic kidney disease globally and remains a major cause of end-stage renal disease (ESRD) [6]. Approximately 40% of diabetic patients develop some features of DKD, leading to a major healthcare burden [7]. The molecular mechanisms underlying the development of DKD have been widely explored but the therapeutic strategies for DKD are still limited [8]. The pathogenic role of senescence in DKD and treatments that target this process are gaining increasing attention [9]. Here, we review the current understanding of senescence in DKD and summarize the possible therapeutic interventions relevant to senescence in the field.

## Characteristics of cellular senescence

### Senescence-associated β-galactosidase (SA-β-Gal)

SA-β-Gal activity was reported to be a biomarker for senescent cells in culture and *in vivo* approximately 30 years ago [10]. The enzymatic activity of SA-β-Gal at pH 6.0 can be detected in senescent cells, but not in most non-senescent, quiescent or terminally differentiated cells, whereas acidic β-Gal activity can be detected in all cells at pH4.0 [10] SA-β-Gal activity originates from lysosomes and the enzyme is the classical lysosomal β-D-galactosidase, encoded by the gene *GLB1* [11]. Senescent cells have low activity of β-Gal as a result of knockdown of *GLB1* [11]. Interestingly, lysosomal β-Gal activity can be detected in all non-senescent cells at pH 4.0, the optimal condition for the enzyme. In a suboptimal environment at pH6.0, the activity of β-Gal is usually not detectable in most non-senescent cells. In senescent cells, lysosomal biogenesis is increased leading to an increased number of lysosomes and an increased abundance of lysosomal enzymes, including the lysosomal enzyme β-Gal. The enzymatic activity of the abundant lysosomal β-Gal in these senescent cells becomes detectable under the suboptimal condition at pH 6.0 [12–14]. The β-D-galactose residues in β-D-galactosides are the substrates of β-Gal [8]. To detect the activity of β-Gal in an assay, the X-gal chromogen (5-bromo-4-chloro-3-indoyl β-d-galactopyranoside) is the most popular chemical used which serves as an exogenous substrate, and can be cleaved into galactose and 5-bromo-4-chloro-3-hydroxyindole. 5-bromo-4-chloro-3-hydroxyindole is then oxidized to a blue product, 5,5′-dibromo-4,4′-dichloro-indigo, which reflects the activity of β-Gal activity in that assay [15,16].

However, the detected activity of β-Gal at pH 6.0 is not specific to senescent cells. The increased lysosome biogenesis due to autophagy activation can also lead to increased β-Gal activity [17,18]. The staining is also increased in immortal cells when they reach high cell density [19]. The β-Gal activity at pH 6.0 is also detectable in tissue-resident macrophages [20]. Furthermore, the osteoclasts in bone marrow show a high level of β-Gal activity, regardless of the staining pH [21]. Therefore, this marker, despite being useful, cannot be used alone as a definitive marker to reliably define cellular senescence. Thus, it is recommended that several other hallmarks of senescence should be used in combination with SA-β-Gal to reliably identify senescent cells.

### Cell cycle arrest

Irreversible cell cycle arrest is an important feature of senescent cells, which has attracted researchers’ attention for >30 years [22,23]. The p53-p21- retinoblastoma protein (RB) pathway plays an important role in regulating the cell division cycle [24]. p53, encoded by the *TP53* gene, is a tumor suppressor, whose loss of function mutations are associated with malignant proliferation [25]. The protein p53 is a powerful transcription factor regulating transcriptional expression of multiple genes and biological processes in response to cellular stresses such as oncogene activation and DNA damage [26]. p21 is encoded by the cyclin-dependent kinase inhibitor 1A (*CDKN1A*), also known as wildtype activating factor-1 (WAF1) or cyclin-dependent kinase inhibitory protein-1 (CIP1) [27]. The p21 protein interacts with cyclin-dependent kinase (CDK), inhibiting CDK activity and acting as a negative cell cycle regulator [28,29]. Retinoblastoma protein (RB), encoded by *RB1*, belongs to a family comprising three members including RB, retinoblastoma-like 1 (RBL1 or p107), and retinoblastoma-like 2 (RBL2 or p130) [30]. RB is a critical regulator inhibiting the G1/S transition, whose deficiency is associated with many malignant tumors including retinoblastoma, prostate cancer, lung cancer, and breast cancer [30]. RB modulates the cell cycle via interactions with the early region 2 binding factors (E2Fs), a transcription factor family that serves as a positive regulator for cells to progress to the S phase [31,32]. In detail, hypo-or monophosphorylated RB forms a complex with E2Fs, attenuating the function of E2Fs as a cell division promoter [31,33]. However, the highly activated CDK would hyperphosphorylate RB, dissociating the RB/E2Fs complex thus leading to E2Fs activation [31]. Collectively, p21 is activated by p53 at the transcriptional level, followed by the inhibition of CDKs, such as CDK1 and CDK2, by the p21 protein [34]. The inhibition of CDKs results in hypo- or monophosphorylation of RB and the inactivation of E2Fs, ultimately leading to cell cycle arrest [24]. Apart from the p53-p21-RB pathway, another critical CDK inhibitor named p16, encoded by cyclin-dependent kinase inhibitor 2A *(CDKN2A)*, is also involved in inhibiting cell cycle progression [35], and its up-regulation is associated with cellular senescence. Indeed, tissue accumulation of p16 positive cells accelerates organ aging and elimination of these cells delays ageing-associated disorders in vivo [36,37]. p16 belongs to the inhibitor of cyclin-dependent kinase 4 (INK4) family, which consists of four members including p16/INK4A, p15/INK4B, p18/INK4C and p19/ INK4D [38]. p16 binds to CDK4/6 and inhibits their activity, resulting in the hypo- or monophosphorylation of RB and subsequent abrogation of E2F function [39]. Transcriptional regulation of p16 is mediated by two complexes comprised of multiple proteins, such as B-cell-specific Moloney murine leukemia virus integration region 1 (BMI-1) and Enhancer of zeste homolog 2 (EZH2) [39]. The two protein complexes negatively regulate the expression of p16 by methylating its gene promoter [39].

In the 1990s, the up-regulation of p53, p21, and p16 in senescent cells was reported to be useful biomarkers for cellular senescence [40–43]. Generally, the p53-p21 pathway is activated upon DNA damage response (DDR) and p16 can be induced by oncogenic activation and by various other stressors [44]. Interestingly, it was reported that in early senescent cells, p21 was increased but p16 remained at a low level. However, in late senescent cells, the level of p21 could return to a nearly normal level but p16 accumulated to a high level [45]. Thus, the p53-p21 axis plays a role in regulating senescence in the early stage and might be important for senescence induction [46], whereas p16 is necessary for the maintenance of cellular senescence during the relatively late stage [45,46]. Collectively, p53-p21 and p16 are responsible for cell cycle arrest jointly or separately according to the stressor and cell type [47].

### Senescence-associated secretory phenotype (SASP)

SASP was reported in senescent cells >20 years ago when overexpression of certain cytokines such as interleukin 1 α (IL-1α), and intercellular cell adhesion molecule (ICAM) were observed in senescent cells [22]. However, the concept of SASP wasn’t clearly defined until 2008 [48], when Campisi and colleagues studied the molecules secreted by different senescent cells. They initially found that all the different cell populations showed a similar feature upon senescence induction, e.g. having high levels of secreted inflammatory cytokines and chemokines, immunomodulatory cytokines, and growth factors, with increased expression of inflammatory cytokines such as IL-6 and IL-8 considered the core feature of the SASP [48]. However, more extensive studies subsequently showed that SASP composition could be heterogeneous, and affected by the cell type and the nature of the stresses [49]. In fibroblasts, senescent cells induced by ionizing radiation were found to secrete 343 SASP proteins [50]. The most highly up-regulated proteins in the senescent fibroblasts included inflammatory cytokines, hemostasis-related factors and extracellular matrix (ECM) proteins [50]. In another study, quantitative unbiased proteomic analysis of secreted soluble proteins and exosomes in the medium of cells treated with three different senescence inducers revealed that hundreds of proteins were significantly changed in senescent fibroblasts and renal epithelial cells [51]. That study demonstrated that chemokine C-X-C motif ligands (CXCLs), matrix metalloproteinases (MMPs), and tissue inhibitors of metallopeptidase (TIMPs), were frequently detectable SASP molecules [51]. It was also observed that there were fewer elevated proteins detected in renal epithelial cells than in fibroblasts, indicating the distinct composition of the SASP in different cell populations [51].

### DNA damage response (DDR)

Stimuli such as chemotherapeutic drugs, chemicals, γ-irradiation, ultraviolet radiation, and endogenous oxidative damage, can cause single-strand or double-strand breaks in chromosomal DNA molecules, which trigger the DDR in affected cells [52,53]. The protein kinases Ataxia Telengectasia Mutated (ATM) and Ataxia Telangiectasia and RAD3-related (ATR), belonging to the phosphatidylinositol 3-kinase-like protein kinases (PIKKs) family, are key sensors of DNA damage or genotoxic stress [54,55]. ATR/ATM phosphorylates checkpoint kinase 1/checkpoint kinase 2 (CHK1/CHK2)which can phosphorylate p53. The phosphorylation of p53 induces accumulation of p53 in the cells, which transcriptionally up-regulates p21, leading to cycle arrest and cellular senescence [56]. DNA damage is known to induce not only cellular senescence but also apoptosis, with speculation that prolonged moderate DNA damage leads to cellular senescence, while severe DNA damage results in apoptosis [57].

The DDR is both an activator and a feature of senescence. Most of the stimuli activating senescence could affect DNA directly or indirectly. For example, telomere shortening during replicative senescence leads to DNA breaks [58]. In oncogene-induced senescence, cellular hyperproliferation triggered by oncogene activation is accompanied by accumulated genomic damage, which will activate the DDR [56]. Therefore, the activation of DDR signaling can serve as a marker to identify cells in the senescent state. There are many DNA damage response related proteins, which have been extensively studied, such as γH2AX, ATM, p53-binding protein 1 (53BP1) and mediator of DNA damage checkpoint protein 1 (MDC1) [55,59]. Phosphorylation of Serine 139 of histone H2AX (γH2AX) has been recognized as the activation signal of the DDR [60]. When DNA double-strand breaks (DSB) occur, the PI3K-like kinases such as ATM are activated and phosphorylate H2AX [61]. γH2AX foci were found in the region near the DSB site shortly after the cells were exposed to DNA damage stimuli [61]. Increased deposition of γH2AX has been widely used as a marker of DDR or a genotoxic stress marker in research [62,63]. Similarly, increased levels of 53BP1, MDC1 and the ATM/ATR/CHK1/CHK2/p53 cascade can also be detected to characterize the cellular senescence state [64,65].

### Senescence-associated heterochromatin foci (SAHF)

Heterochromatin is a modified and condensed chromatin structure where some proliferation-promoting genes are included and transcriptionally inactivated. Facultative heterochromatin formation is considered to be the key mechanism for X inactivation in female cells where one of the two X chromosomes is transcriptionally inactivated. In 2003, Narita and colleagues reported that the formation of facultative heterochromatins can be stimulated by senescence inducers in cultured human diploid fibroblast cells (IMR-90 cell line). In these induced senescent cells, the heterochromatins were shown to contain E2F target genes of the proliferation promoting pRB/E2F pathway as well as binding of a histone H2A variant, macroH2A and common heterochromatin marker proteins such as heterochromatin proteins 1 (HP1) and Histone 3 with its Lysine [9] residue methylated (Me-K^9^-H3) [66]. This finding is consistent with the previous observation that senescent cells have extensive chromatin remodeling [67]. The unique domains of the transcriptionally silent foci observed in senescent cells are known as SAHF. SAHF can be detected by dense 4′,6′-diamidino-2-phenylindole (DAPI) staining and visualized by dense puncta staining patterns enriched and colocalized for repressive chromatin marks such as trimethylated histone H3 Lys9 (H3K9me3), macroH2A and HP1 [66,68,69]. However, SAHF is not always obviously detected in senescent cells induced by different senescence stimuli. For example, oncogene-induced senescence by exogenous expression of oncogenic Ras is associated with stronger SAHF staining, when compared with other inducers, such as ionizing radiation and H_2_O_2_ treatment [70].

### Oxidative stress

Oxidative stress is a senescence driver and also a feature of cellular senescence. Reactive oxygen species (ROS) can promote senescence by inducing mitochondrial DNA (mtDNA) damage and activating the p53, p21, and p16 pathways [71]. In fact, ROS accumulation also results in the oxidization of mitochondrial proteins and enzymes, which further impairs mitochondrial function [72]. Mitochondrial dysfunction can lead to a decrease in the efficiency of oxidative phosphorylation and more ROS production, which, together, can further accelerate cellular senescence [73].

H_2_O_2_ can induce a senescent phenotype in cultured cells and has been widely used as a cellular senescence inducer [74–76]. In contrast, antioxidants can inhibit cellular senescence by reducing ROS levels [77]. The activation of antioxidant signaling pathways such as the Nrf2 pathway is associated with the amelioration of senescent phenotypes [78]. Therefore, the detection of ROS is a useful parameter to reflect the cellular status of senescence.

### Premature senescence in DKD

The accelerated cellular senescence in a diabetic environment has been demonstrated in both animal models and in human kidney biopsy samples. p21 is persistently induced in kidneys from STZ-induced diabetic mice and db/db mice, at both the mRNA and protein levels [79]. Other senescence markers including SA-β-Gal staining, γH2AX, and p16 are also observed, supporting the presence of premature senescence in the kidneys of diabetic mice [79,80]. Diabetic Akita mice show higher levels of senescence-associated gene expression, higher oxidative stress, and more mitochondrial DNA damage when compared with wild-type counterparts [81]. The bradykinin B2 receptor (B2R), a G-protein coupled receptor (GPCR), involved in neurodevelopment, neuroprotection, blood pressure regulation, and inflammation control [82], is reported to inhibit oxidative stress- and high-glucose-induced senescence, via the p53 and RB pathways [83,84]. Indeed, Akita mice with a mutation at the B2R locus (Bdkrb2–/–Ins2Akita/+) have a more severe senescence phenotype in the kidney [81]. By contrast, genetic deletion of p66^ShcA^, an adaptor protein mediating the intracellular signal transduction of GPCRs, can alleviate the aging phenotype in the kidneys of Akita diabetic mice [85]. Indeed, p66^ShcA^ has been shown to increase the levels of ROS in cells treated with high glucose and promote oxidative stress in DKD mice [86,87].

Indeed, oxidative stress as a result of increased intracellular accumulation of ROS has been shown to be a critical pathological stimulus in DKD. NADPH Oxidase 4 (NOX4) is a major NOX isoform expressed in the mouse kidney, and it is the key enzyme producing ROS in the kidney in diabetic mice. Previous studies have shown that either genetic deletion or pharmacological inhibition of NOX4 reduces renal ROS levels leading to attenuation of experimental DKD, including key profibrotic and proinflammatory parameters [88]. On the other hand, transgenic expression of the human Nox5 gene in mouse kidneys, which is otherwise absent in rodents, further increases renal ROS levels and consequently exacerbates DKD in mice [89]. In the latter study, renal expression of p21, one of the key cellular senescence markers, and renal levels of ROS were concurrently increased in diabetic mice, with these changes further increased by transgenic expression of human NOX5 [89]. Both NOX4 and NOX5 are expressed in the human kidney and their expression levels are elevated in DKD. These experimental data support the view that approaches to reduce oxidative stress are effective to confer renoprotection in DKD, likely as a result of reduced oxidative stress-induced cellular senescence.

In human samples, SA-β-Gal staining and p16 expression are increased in Type 2 diabetic nephropathy biopsies compared with normal age-matched control tissues, supporting the presence of increased cellular senescence in DKD [90]. Furthermore, the markers of senescence are more pronounced in tubular cells than in glomeruli [90]. Interestingly, glomerular p16 expression was found to be associated with proteinuria, while tubular p16 was associated with key risk factors for diabetes, such as body mass index, LDL cholesterol, and HbA1c [90]. Increased γH2AX staining is also reported in the kidney from DKD patients [79]. Mechanistically, high glucose has been shown to induce senescence in tubular epithelial cells, glomerular mesangial cells, endothelial cells, and immortalized podocytes [91–94].

**Figure 1:**
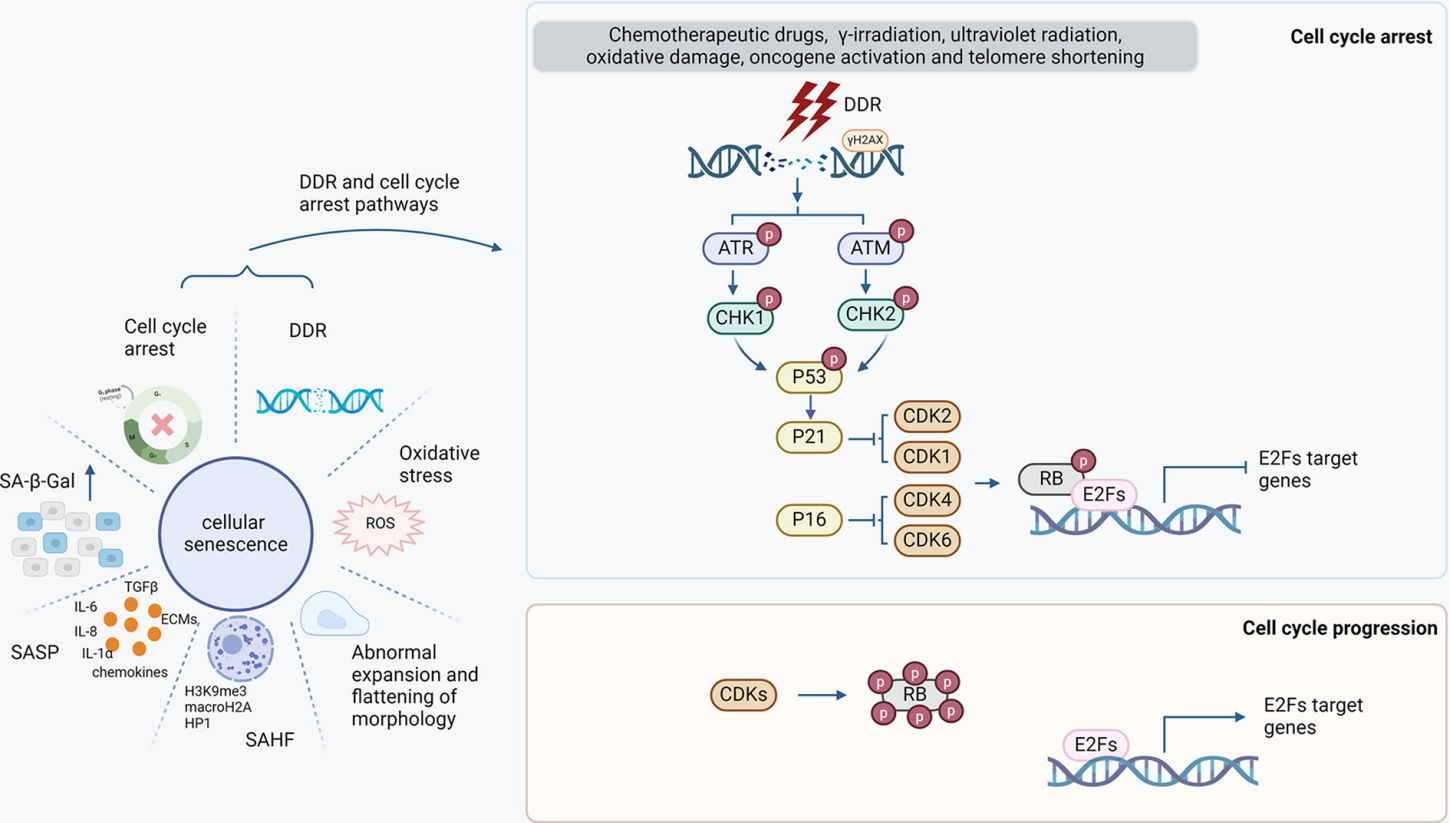
Characteristics of cellular senescence Left panel: Cellular senescence is characterized by increased activity of SA-β-gal, SASP, cell cycle arrest, DDR, oxidative stress, abnormal expansion and flattening of cell in morphology as well as SAHF. SASP can be identified by detection of secreted chemokines, ECMs, TGFβ, and inflammatory cytokines such as IL-1α, IL-6, and IL-8. SAHF is presented as DNA foci in the nucleus that are colocalized with enriched H3K9me3, macroH2A, and HP1. Oxidative stress is driven by excessive ROS accumulation. There is an association between DDR and cell cycle arrest. Right and upper panel: Exogenous or endogenous stress causes DNA damage, accompanied by up-regulation of γH2AX. ATR and ATM are DNA damage sensors, that can phosphorylate CHK1 and CHK2, respectively. Activated CHK1/2 phosphorylate p53, resulting in p53 up-regulation, which in turn up-regulates p21, the CDK inhibitor. The inhibition of CDK1/2 by p21 and the inhibition of CDK 4/6 by p16 result in hypo- or mono-phosphorylation of RB, promoting RB-E2F complex formation, leading to inhibition of the proliferation-promoting function of E2F and ultimately cell cycle arrest. Right lower panel: CDKs promote hyper-phosphorylation of RB, supporting RB dissociation from E2F and cell cycle progression.

Taken together, these biological characteristics are observed in senescent cells derived from various cell types and/or in various contexts (Figure 1). Since some of these changes also occur and can be detected in other biological processes unrelated to cellular senescence, detection of multiple biomarkers for these characteristics is essential in order to identify and characterize senescent cells in DKD. It should be appreciated that the potential pathological roles of these cellular senescence related biological characteristics are not yet fully elucidated. Possible pathogenic roles of some of these biological characteristics and related biomarkers in DKD are summarized in Table 1.

**Table 1:** Characteristics of the senescence biomarkers and their possible pathogenic role

Characteristics of cellular senescence	Detected marker	Possible pathogenic role in DKD
Increased SA-β-Gal activity	Stain with X-gal chromogen	Pathogenic role unknown; Reflection of increased lysosomal biogenesis; Reflection of activated autophagy.
Cell cycle arrest	p53, p21, p16,	Cell cycle arrest; Inhibiting cell proliferation; Impairing reparative capacity.
SASP	Cytokines, ICAM, ECM, CXCLS	Promoting inflammation; Promoting fibrosis.
DDR	γH2AX, ATM, 53BP1	Inhibiting cell proliferation; Impairing reparative capacity.
SAHF	H3K9me3, macroH2A, HP1	Affecting gene expression; Inhibiting cell proliferation; Impairing reparative capacity; Reflection of DNA damage; Reflection of telomere shortening/stress.
Oxidative stress	ROS	Damaging cellular molecules; Increasing cell death; Promoting inflammation; Promoting fibrosis

## Biomarkers of senescence in DKD

Urinary p21 is detectable in samples from DKD patients, but not in healthy controls or in patients with other diseases with normal kidney function [79]. The association of levels of urinary p21 with an increasing grade of CKD using KDIGO criteria has also been confirmed in a large cross-sectional cohort [79]. Plasma Activin A, a marker of SASP, is quantitatively associated with senescence and kidney fibrosis in DKD. Compared with individuals without diabetes or CKD, patients with DKD and reduced kidney function have significantly increased levels of plasma Activin A [95]. Some urinary inflammatory cytokines such as IL-6, IL-10, the molecules present in the SASP, are also proposed to be indicators of kidney injury in DKD [96]. Although the relationships among these senescence markers and kidney damage are reported as described above, all these studies show no direct evidence of the association between the levels of these urinary senescent molecules and the degree of senescence in the kidney.

## The regulation of senescence in DKD

### Redox pathways

Oxidative stress and the associated signaling pathways play important roles in regulating cellular senescence in DKD. Glomerular endothelial senescence could be driven by M1 macrophages and is dependent on intracellular ROS [94]. As mentioned above, NADPH oxidase (NOX) is a significant contributor to oxidative stress in DKD. NOX1 is reported to promote premature senescence in DKD by modulating the p38/p27 signaling pathway and activating PKCα/β [80]. The kelch-like ECH-associated protein 1 (KEAP1)/nuclear factor erythroid 2-related factor 2 (NRF2) pathway is a powerful modulator of redox balance, which also plays an important role in aging [97]. Pyrroloquinoline quinone (PQQ), a redox coenzyme, can inhibit oxidative stress and senescence in HK-2 cells in a high glucose environment by affecting the KEAP1/NRF2 pathway [98]. The lower senescence is associated with enhanced NRF2 translocation to the nucleus and increased levels of certain downstream antioxidants [98]. β-Hydroxybutyrate is one of the ketone bodies derived from fatty acid metabolism and used as an energy source when glucose cannot be efficiently used. β-Hydroxybutyrate has been shown to inhibit ROS generation, lipid peroxidation and protein oxidation [99]. Moreover, β-hydroxybutyrate can mitigate senescence in the kidney in STZ-induced diabetic mice as a result of NRF2 restoration, which has been confirmed *in vitro* in immortalized mouse podocytes cultured in high glucose media containing TGFβ1 [93]. Indeed, NRF2 is repressed in podocytes in diabetic kidney by glycogen synthase kinase 3β (GSKβ), a redox-sensitive transducer [100].

### Autophagy

Autophagy is an important cellular process that degrades and reuses dysfunctional cellular components to regenerate new and functional cellular components. The effect of autophagy on cellular senescence appears to be complex [101]. Autophagy inhibition can either delay or promote cellular senescence, depending on cell type, senescence stimulus used, and the timing and duration of autophagy inhibition [101,102]. Inhibition of autophagy with 3-methyladenine (3-MA) contributes to advanced glycation end product (AGE)-induced senescence in mesangial cells [103]. Autophagy is associated with the alleviation of senescence in experimental DKD partially because of the degradation of SASP molecules as shown *in vivo*, and in HK-2 cells [104]. Mitophagy, an autophagy process to remove damaged mitochondria, has also been shown to affect cellular senescence in the context of DKD. Inhibition of mitophagy with Mdivi-1 (mitochondrial division inhibitor 1) results in enhanced senescence in renal tubular epithelial cells (RTECs) treated with high glucose, while a mitophagy agonist Torin1 reduces cellular senescence markers [91]. Enhancing mitophagy and mitochondrial function by overexpressing yeast mitochondrial escape 1-like 1 (YME1L), a metalloprotease localized to the inner mitochondrial membrane with activity to maintain mitochondrial integrity protects RTECs against senescence in DKD mice [105]. However, the molecules and pathways mediating the effect of autophagy in influencing cellular senescence in DKD remain to be fully elucidated. Interestingly, the autophagic degradation of GATA4 mediated by the autophagic receptor protein p62 is considered to be a possible mechanism [106]. The accumulation of GATA4 in human fibroblast IMR-90 cells activates SASP and promotes cell cycle arrest, which could be eliminated by autophagy [106]. Indeed, GATA4 has been found to promote senescence in DKD mice [107].

### Endoplasmic reticulum (ER) stress

The ER is an important cellular enclosed compartment where membrane proteins and secretory proteins are synthesized by the ribosomes attached to the ER (rough ER), and then modified and properly folded within the ER to become functional proteins which are exported from the ER. The ER is also responsible for the synthesis of lipids, phospholipids and cholesterol as well as secretion of steroid hormones. Therefore, the ER is responsible for the modification, folding and trafficking of proteins. Misfolded or unfolded proteins cannot be exported and are accumulated within the ER, causing ER stress [108]. ER stress has been shown to affect multiple cell types and plays a vital role in the progression of DKD [109]. Importantly, ER stress might be involved in the progression of premature senescence in DKD [110,111]. Colocalization of the ER stress marker glucose-regulated protein 78 kDa (GRP78) and the cellular senescence marker p16 or p21 was confirmed in proximal tubular epithelial cells (PTECs) [110,111]. Furthermore, inhibition of ER stress by 4-phenylbutyrate (4-PBA) alleviated premature senescence induced by AGEs, and ER stress inducers were shown to increase the proportion of senescent PTECs *in vitro* [110,111].

Like mitochondria which are considered to be the major source of ROS, the ER is also a significant contributor and is estimated to produce ∼25% of total ROS [112]. Indeed, ROS are natural by-products of the key biological process of protein folding within ER. ER stress interacts with oxidative stress, which might be the critical mechanism for cell fate regulation [113]. The specific role of ER stress networks in cellular senescence modulation remains to be fully defined.

### Transforming growth factor-β (TGFβ)

**Figure 2:**
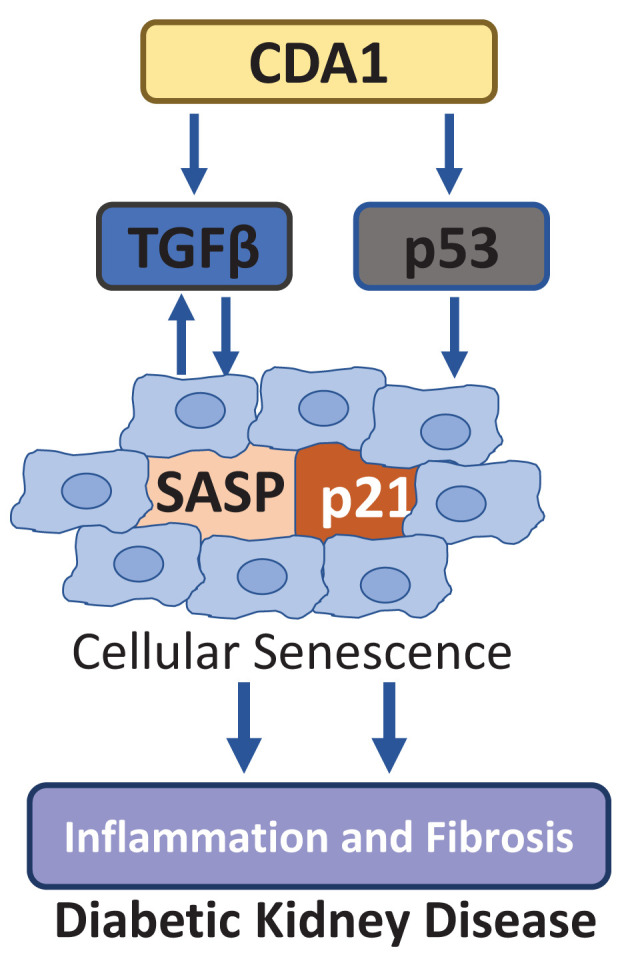
Model of CDA1’s action in promoting cellular senescence in diabetic kidney via enhancing TGFβ signaling and activating p53/p21 pathways CDA1 has been shown to synergistically enhance TGFβ signaling in the context of diabetes [130,124] and to induce p53 in DNA damage response [127,128]. Indeed, CDA1 overexpression leads to arrest of cell cycle, as a result of CDA1-induced p21 up-regulation, which can be inhibited by p53 siRNA knockdown, ERK MAPK inhibitors or TGFβ type I receptor inhibitor. Genetic deletion of CDA1 or pharmacological inhibition of CDA1 in diabetic mouse has been shown to attenuate renal inflammation and fibrosis, the key features of DKD [129,131]. These findings support the view that CDA1 synergistically enhances profibrotic TGFβ signaling and induces p53 leading to cellular senescence in diabetic kidney, which further stimulates inflammation and fibrosis via SASP.

The evidence for the regulation of senescence by the TGFβ signaling pathway was reported approximately three decades ago [114–116]. Mechanistically, TGFβ promotes senescence by inducing cell cycle arrest-related genes and SASP molecules via Smad and non-Smad pathways, albeit this has only been shown in cancer cell lines and non-kidney cell lines [117]. Furthermore, TGFβ1 is a member of SASP. TGFβ1 levels are associated with levels of senescence markers such as p21, SASP molecules and oxidative stress in DKD [118,119]. TGFβ has been shown to be a critical pathogenic growth factor in DKD and anti-TGFβ approaches have been shown to attenuate diabetes induced renal injury in animal models of DKD [120–123]. Furthermore, cell division autoantigen 1 (CDA1), a molecule linked to TGFβ levels and action [124], was initially shown to up-regulate p21 expression via the p53 pathway leading to cell cycle arrest in HeLa cells [125–127], and in DNA damage response in various cancer cells [128], was subsequently found to synergistically enhance TGFβ signaling in HK-2 cells and to promote diabetes associated renal injury in DKD [129–131]. In those studies, a molecular approach to knockdown CDA1 in cells as well as genetic and pharmacological approaches to knockout the CDA1 gene or inhibit CDA1’s activity *in vivo* are effective attenuating TGFβ signaling and ameliorating kidney injury in animal models of diabetes. These findings support the view that the CDA1/TGFβ axis plays a key pathological role in DKD, probably as a result of promoting cellular senescence thus compromising tissue repair in the kidney upon injury by diabetes, as well as by enhancing the profibrotic process leading to tissue scar formation, known as fibrosis, at a later stage of disease (Figure 2).

There are other proteins reported to influence senescence in DKD. For instance, protease-activated protein C is reported to reverse senescence in DKD by reducing tubular p21 expression [79]. In addition, valproic acid can mitigate senescence in STZ-induced DKD mice [132], and deletion of the C5a receptor attenuates cellular senescence and the senescence-associated secretory phenotype in mice with DKD [132].

## Impacts of senescence in DKD

The SASP contains pro-inflammatory cytokines, such as IL-1β, IL-6, TNFα, and MCP-1 [48], and pro-fibrotic molecules such as TGFβ1, resulting in chronic inflammation and fibrosis. The pro-inflammatory cytokines can cause sustained inflammation which contributes to the pathogenesis and progression of DKD [133]. The pro-fibrotic molecules, such as TGFβ1, are powerful drivers of kidney fibrosis [134] Indeed, concurrent senescent cell accumulation and kidney fibrosis have been widely observed in mice upon renal injury [135–137].

**Figure 3:**
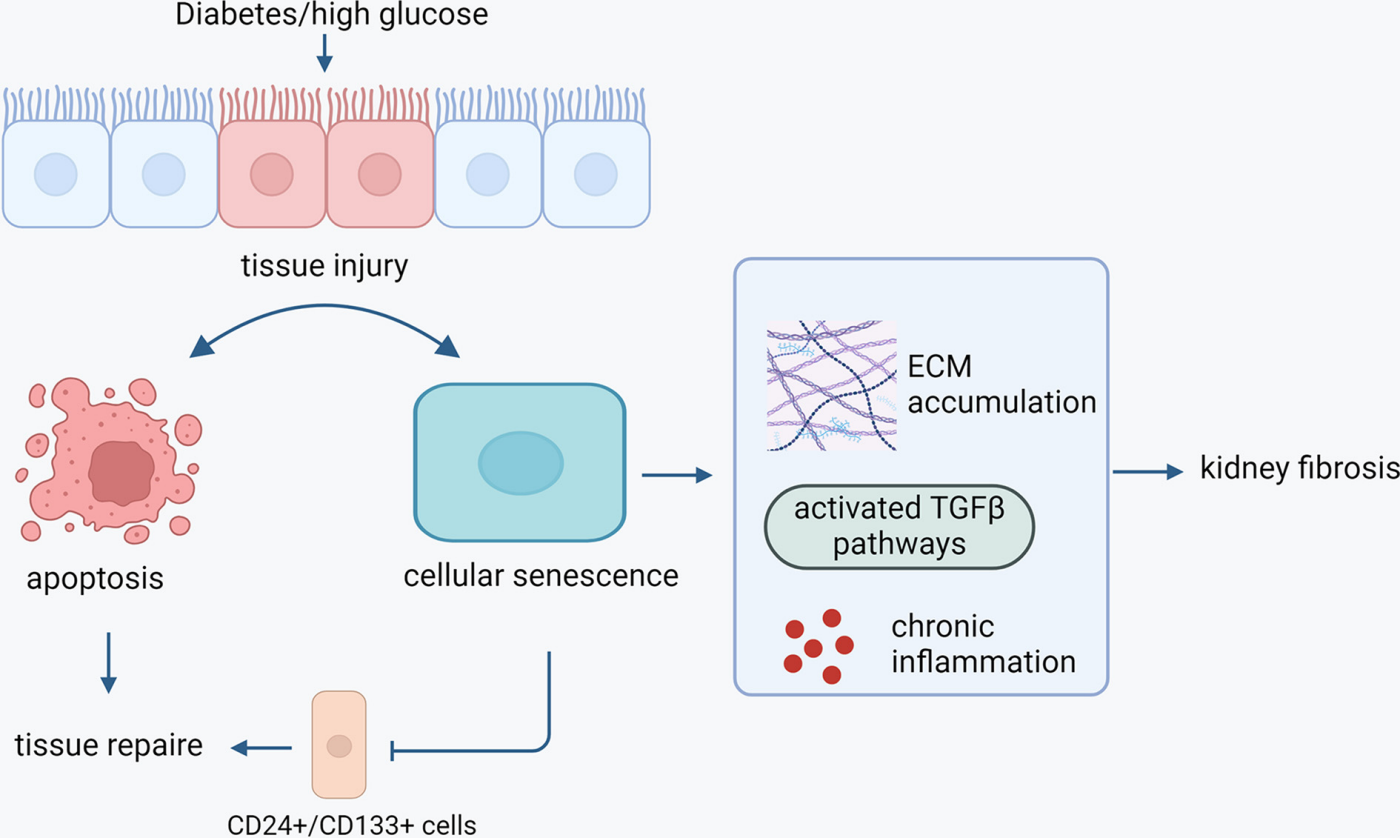
Impacts of cellular senescence in DKD Diabetic or high glucose environment promotes tissue injury, resulting in cellular senescence and cell death such as apoptosis. Renal CD24^+^/CD133^+^ cells with self-renewal potential participate in the repair process upon injury. However, in diabetes, increased cellular senescence compromises their proliferation and senescent cells secret proinflammatory and profibrotic molecules in the kidney, leading to maladaptive repair and increasing loss of functional kidney tissue. Furthermore, senescent cells secrete pro-inflammatory cytokines and pro-fibrotic molecules, which further damage kidney tissue accompanied by sustained inflammation and fibrosis, ultimately leading to loss of renal function or kidney failure as a result of increasingly accumulated unrepaired tissue damage.

Proximal tubules with senescent phenotypes show maladaptive repair after injury [138]. Proximal tubules appear to be the source of specialized epithelial cells with co-expression of the stem cell markers CD133 and CD24, which are able to differentiate into various renal cell populations [139,140]. The CD24^+^/CD133^+^ scattered tubular-like renal cells (CD24^+^/CD133^+^STC) are single cells scattered throughout the tubules and have self-renewal potential, playing an important role in renal recovery after acute injury [141,142]. Indeed, it was demonstrated that infusion of renal CD24^+^/CD133^+^ STCs attenuated ischemia-induced renal injury with improved renal parameters such as less hypoxia, reduced fibrosis, decreased inflammation and diminished capillary loss in mouse, while senescent renal CD24^+^/CD133^+^ STCs had impaired reparative capacity [143]. Therefore, cellular senescence plays a key role in impairing repairing capacity of renal cells, leading to disease progression (Figure 3).

Senescent cells appear to equip themselves with anti-apoptotic capability, via regulation of the Bcl-2 family of proteins [144]. Apoptosis resistance contributes to the accumulation of senescent cells in the kidney due to the ineffective elimination of these senescent cells [145,146]. In DKD, Decoy receptor 2 (DCR2), a p53 target gene influencing chemosensitivity by inhibiting the pro-apoptotic activity of the tumor necrosis factor-related apoptosis-inducing ligand (TRAIL), was recently shown to interact with peroxiredoxin 1 (PRDX1) to induce senescence of RTECs, which play an important role in promoting renal fibrosis in DKD [137]. DcR2 was shown to induce senescent RTECs with an apoptosis-resistant phenotype by interacting with GRP78, a major ER chaperone protein playing a key role in regulating the Unfolded Protein Response in ER stress [147]. Taken together, these studies show that increased accumulation of senescent cells is associated with inflammation and fibrosis as well as the consequent loss of renal function in the diabetic kidney [37,79,148].

## Treatments to target cellular senescence

### Anti-senescence effects of glucose-lowering medications used in T2D treatments

Some therapies that aim to improve glycemic control also show renoprotection in patients with Type 2 diabetes (T2D) [7]. Interestingly, the glucose-lowering drugs with renal protective effects are able to mitigate cellular senescence.

Metformin is one of the most popular oral glucose-lowering agents and is often the first drug used in T2D. In DKD, metformin has been shown to decrease oxidative stress and augment autophagy [149]. It can alleviate inflammation and retard fibrosis via regulation of the NFκB and TGFβ pathways [150]. It reduces senescence in HK-2 cells treated with high glucose and in db/db mice via the MBNL1/miR-130a-3p/STAT3 pathway or by regulating the expression of E2F1 [151,152]. Indeed, metformin has been proposed as a possible anti-aging drug with multiple target pathways [153]. In several human fibroblast cell lines (WI-38 cells, IMR‐90 cells, and IDH 4 cells), this drug can decrease markers of senescence, including p16, p21, IL-6, and IL-8 [154]. In murine olfactory ensheathing cells, metformin was shown to inhibit the NFκB pathway and decrease various cellular senescence markers such as SA-β-Gal activity, oxidative stress as well as inflammatory cytokines [155]. In addition, metformin down-regulates senescence and SASP via the Nrf2 and AMPK pathways [156,157].

Dapagliflozin, a sodium-glucose cotransporter-2 inhibitor (SGLT2i), can mitigate the senescence phenotype induced by high glucose in primary human RTECs [119]. SGLT2i exposure for 8 weeks is able to decrease senescence markers in the kidneys of db/db mice, including the protein levels of 53BP1, p16, p21, γH2AX, as well as SASP [158]. However, in another study, treatment with SGLT2i alone for 6 weeks failed to reduce the levels of p21 and γH2AX in STZ-induced diabetic mice, despite a significant reduction in UACR [79]. The reason for the inconsistent results might be different animal models and different treatment protocols used, such as dosage and treatment durations. The latter study also showed a trend toward decreased senescence markers, although the differences were not statistically significant [79].

Glucagon-like peptide-1 (GLP-1) receptor agonists are also renoprotective in diabetic patients [159]. Dulaglutide, a GLP-1 receptor agonist, has been reported to alleviate high-glucose-induced senescence-related gene expression and protect against diabetic retinopathy [160]. Since diabetic retinopathy and DKD are both microvascular complications of diabetes [161], it is postulated that these drugs can also alleviate senescence in DKD.

Dipeptidyl peptidase 4 (DPP-4) inhibitor is a new class of glucose-lowering medicines. Although no renal protection was found in RCTs according to the result of the composite renal outcome, DPP-4 inhibitors could partially delay the progression of albuminuria [162–164]. Recently, researchers found that DPP-4 on the surface of senescence-associated extracellular vesicles appears to hinder the internalization of the vesicles by HeLa cells, which implicated a possible role of DPP-4 in mediating the effect of senescent cells on other cells in proximity [165]. However, the role of DPP-4 inhibitor in influencing cellular senescence and/or cellular senescence-associated pathological effects in DKD needs to be elucidated by future studies.

### Senotherapy

Senotherapy is a treatment strategy to specifically target senescent cells (SNCs) in order to restore tissue integrity by mitigating the adverse effects associated with cellular senescence [166]. This strategy focuses on the blockade of SASP and the clearance of SNCs, the latter approach known as senolysis or senolytics [166]. Senolytics eliminate SNCs by targeting the pathways that are differentially activated in SNCs but not in non-SNCs, such as the anti-apoptotic pathway [166,167].

One of the classical senolytics is the combination of Dasatinib and Quercetin (D + Q). Dasatinib, a tyrosine kinase inhibitor, was approved for the treatment of leukemia by the FDA in 2006 [168]. Quercetin is a natural flavonoid targeting anti-apoptosis network members including BCL-xL and HIF1α [169]. Such an approach has been shown to have multiple beneficial effects, such as reduced inflammation and being an anti-oxidant. Kirkland's team has for the first time used D+Q as a therapy for senescence [170]. They creatively explored the Senescent Cell Anti‐Apoptotic Pathway (SCAP) networks to identify a potential drug target to eliminate SNCs. First, they confirmed that the inhibition of the SCAP network by siRNA could effectively promote apoptosis in SNCs without toxicity to the non-SNCs. They used bioinformatic approaches to identify D+Q as a promising candidate from 46 potentially senolytic drugs and then demonstrated D+Q as an efficacious agent for SNC elimination in several cell populations. In a Phase II clinical study (NCT02848131), subjects with chronic kidney disease and diabetes were treated with D+Q leading to decreased senescent cells in adipose tissue and skin [171]. The study is still ongoing with the renal outcome yet to be reported.

Furthermore, in a preclinical study, the heat shock protein 90 (HSP90) inhibitor (alvespimycin), a senolytic, reduces kidney senescence in diabetic mice with bilateral renal ischemia–reperfusion injury [172]. As described above, oxidative stress is an important characteristic and inducer of cellular senescence with redox pathways involved in senescence modulation in DKD. Although there is no direct evidence showing that antioxidants retard senescence in DKD, targeting the key ROS producers in the diabetic mouse kidney has been shown to reduce renal injury in DKD as a result of reduced oxidative stress [88,89]. Epigallocatechin gallate (EGCG) is an antioxidant compound in green tea, and has been previously shown to prevent oxidative stress-induced cellular senescence in human mesenchymal stem cells, with this action mediated by Nrf2 [173,174]. The EGCG-Nrf2 axis has been demonstrated to play a key role in reducing renal oxidative stress, inflammation and fibrosis as well as albuminuria in experimental DKD [175].

## Summary and prospects

The incidence of DKD is strongly associated with the duration of diabetes, indicating that a prolonged and complex process of molecular and cellular changes plays a causal role in initiating DKD, a condition of progressive decline in kidney function as a result of progressive tissue injury within the kidney. A number of factors as a result of hyperglycemia and/or insufficient intracellular insulin signaling due to insulin resistance or insulin deficiency, which are the major features of diabetes, have an adverse impact on renal cell health, and can cause cell death in the kidney. Damaged kidney tissue needs to be repaired in order to maintain tissue homeostasis and renal function. Decreased reparative capability is likely to be a key mechanism driving the development and progression of DKD with increasing renal injury. Indeed, increased senescent renal cells are observed in DKD, including CD24^+^/CD133^+^STCs which are responsible for repairing tissue injury in the kidney. These senescent cells not only lose their ability to proliferate in order to participate in the tissue repairing process, but also secrete proinflammatory and profibrotic cytokines which can also significantly impact on other cells leading to inflammation and fibrosis. Therefore, the onset of DKD occurs as a result of concurrent continuous injuring of the kidney by diabetes and cellular senescence induced impairment of tissue repair. This leads to increasing loss of functional renal tissue. Without targeting the senescent cells, the key culprit of the imbalance of tissue homeostasis, continuous loss of kidney tissue and renal function leads to progression of DKD to kidney failure. Interestingly, most of the drugs with renoprotective effects have been shown to counteract the adverse effects of senescent cells in DKD. Furthermore, Senotherapeutics, the therapeutic strategy to specifically target senescent cells is emerging with some agents being evaluated in clinical trials exploring renal outcomes in diabetes.

The detailed regulation of cellular senescence at a mechanistic level in DKD is yet to be fully elucidated. Further research in this field to understand the interaction or crosstalk among the various pathways related to key pathophysiological responses in diabetes, such as oxidative stress, autophagy, ER stress, cell death, and the kidney repair process, is warranted. In addition, the impact of senescence on various specific renal cell population, such as tubular cells, podocytes, and immune cells, are worthy further study.

Although the underlying mechanisms need further investigation, current therapeutic approaches for senescent cell elimination appear to show kidney benefits. Therefore, an optimized therapeutic strategy to target senescent cells in the kidney represents a novel, promising and potentially effective treatment for DKD patients.

## Data Availability

Data sharing is not applicable to this review paper.

## Open Access

Open access for this article was enabled by the participation of Monash University in an all-inclusive *Read & Publish* agreement with Portland Press and the Biochemical Society under a transformative agreement with CAUL.

## Abbreviations

3-MA: 3-methyladenine
β-Gal: β-galactosidase
AGE: advanced glycation end product
CDA1: cell division autoantigen 1
DCR2: Decoy receptor 2
DDR: DNA damage response
DPP-4: dipeptidyl peptidase 4
DKD: diabetic kidney disease
EGCG: epigallocatechin gallate
ESRD: end-stage renal disease
ER: endoplasmic reticulum
GLP-1: glucagon-like peptide-1
HSP90: heat shock protein 90
PRDX1: peroxiredoxin 1
PTEC: proximal tubular epithelial cell
RTEC: renal tubular epithelial cell
SASP: senescence-associated secretory phenotype
SA-β-Gal: senescence-associated β-galactosidase
SCAP: senescent cell anti‐apoptotic pathway
SGLT2i: sodium-glucose cotransporter-2 inhibitor
SNC: senescent cell
T2D: Type 2 diabetes
TGFβ: transforming growth factor-β
TRAIL: tumor necrosis factor-related apoptosis-inducing ligand

## References

[1] Minot C.S. (1891) Senescence and rejuvenation. J. Physiol. 12, 97.113–192.113 10.1113/jphysiol.1891.sp000369 PMC1514260 16991972

[2] Hayflick L. and Moorhead P.S. (1961) The serial cultivation of human diploid cell strains. Exp. Cell. Res. 25, 585–621 10.1016/0014-4827(61)90192-6 13905658

[3] Sharpless N.E. and Sherr C.J. (2015) Forging a signature of in vivo senescence. Nat. Rev. Cancer 15, 397–408 10.1038/nrc3960 26105537

[4] Calcinotto A., Kohli J., Zagato E., Pellegrini L., Demaria M. and Alimonti A. (2019) Cellular senescence: aging, cancer, and injury. Physiol. Rev. 99, 1047–1078 10.1152/physrev.00020.2018 30648461

[5] Hernandez-Segura A., Nehme J. and Demaria M. (2018) Hallmarks of cellular senescence. Trends Cell Biol. 28, 436–453 10.1016/j.tcb.2018.02.001 29477613

[6] Bikbov B., Purccell C.A., Levey A.S., Smith M., Abdoli A., Abebe M. et al. (2020) Global, Regional, and National Burden of Chronic Kidney Disease, 1990-2017: a systematic analysis for the Global Burden of Disease Study 2017. Lancet (London, England) 395, 709–733 10.1016/S0140-6736(20)30045-3 32061315 PMC7049905

[7] Tuttle K.R., Agarwal R., Alpers C.E., Bakris G.L., Brosius F.C., Kolkhof P. et al. (2022) Molecular mechanisms and therapeutic targets for diabetic kidney disease. Kidney Int. 102, 248–260 10.1016/j.kint.2022.05.012 35661785

[8] Selby N.M. and Taal M.W. (2020) An updated overview of diabetic nephropathy: Diagnosis, prognosis, treatment goals and latest guidelines. Diabetes Obes. Metab. 22, 3–15 10.1111/dom.14007 32267079

[9] López-Otín C., Blasco M.A., Partridge L., Serrano M. and Kroemer G. (2023) Hallmarks of aging: an expanding universe. Cell 186, 243–278 10.1016/j.cell.2022.11.001 36599349

[10] Dimri G.P., Lee X., Basile G., Acosta M., Scott G., Roskelley C. et al. (1995) A biomarker that identifies senescent human cells in culture and in aging skin in vivo. Proc. Natl Acad. Sci. 92, 9363–9367 10.1073/pnas.92.20.9363 7568133 PMC40985

[11] Lee B.Y., Han J.A., Im J.S., Morrone A., Johung K., Goodwin E.C. et al. (2006) Senescence-associated beta-galactosidase is lysosomal beta-galactosidase. Aging Cell 5, 187–195 10.1111/j.1474-9726.2006.00199.x 16626397

[12] Kuilman T., Michaloglou C., Mooi W.J. and Peeper D.S. (2010) The essence of senescence. Genes Dev. 24, 2463–2479 10.1101/gad.1971610 21078816 PMC2975923

[13] Kurz D.J., Decary S., Hong Y. and Erusalimsky J.D. (2000) Senescence-associated (beta)-galactosidase reflects an increase in lysosomal mass during replicative ageing of human endothelial cells. J. Cell Sci. 113, 3613–3622 10.1242/jcs.113.20.3613 11017877

[14] Yang N.C. and Hu M.L. (2005) The limitations and validities of senescence associated-beta-galactosidase activity as an aging marker for human foreskin fibroblast Hs68 cells. Exp. Gerontol. 40, 813–819 10.1016/j.exger.2005.07.011 16154306

[15] Burn S.F. (2012) Detection of β-galactosidase activity: X-gal staining. Methods Mol. Biol. 886, 241–250 10.1007/978-1-61779-851-1_21 22639266

[16] Debacq-Chainiaux F., Erusalimsky J.D., Campisi J. and Toussaint O. (2009) Protocols to detect senescence-associated beta-galactosidase (SA-betagal) activity, a biomarker of senescent cells in culture and in vivo. Nat. Protoc. 4, 1798–1806 10.1038/nprot.2009.191 20010931

[17] Young A.R. and Narita M. (2010) Connecting autophagy to senescence in pathophysiology. Curr. Opin. Cell Biol. 22, 234–240 10.1016/j.ceb.2009.12.005 20045302

[18] Ivanov A., Pawlikowski J., Manoharan I., van Tuyn J., Nelson D.M., Rai T.S. et al. (2013) Lysosome-mediated processing of chromatin in senescence. J. Cell Biol. 202, 129–143 10.1083/jcb.201212110 23816621 PMC3704985

[19] Severino J., Allen R.G., Balin S., Balin A. and Cristofalo V.J. (2000) Is beta-galactosidase staining a marker of senescence in vitro and in vivo? Exp. Cell. Res. 257, 162–171 10.1006/excr.2000.4875 10854064

[20] Bursuker I., Rhodes J.M. and Goldman R. (1982) Beta-galactosidase–an indicator of the maturational stage of mouse and human mononuclear phagocytes. J. Cell. Physiol. 112, 385–390 6813341 10.1002/jcp.1041120312

[21] Kopp H.G., Hooper A.T., Shmelkov S.V. and Rafii S. (2007) Beta-galactosidase staining on bone marrow. The osteoclast pitfall. Histol. Histopathol. 22, 971–976 17523074 10.14670/HH-22.971

[22] Campisi J. (1997) The biology of replicative senescence. Eur. J. Cancer 33, 703–709 10.1016/S0959-8049(96)00058-5 9282108

[23] Rittling S.R., Brooks K.M., Cristofalo V.J. and Baserga R. (1986) Expression of cell cycle-dependent genes in young and senescent WI-38 fibroblasts. Proc. Natl Acad. Sci. 83, 3316–3320 10.1073/pnas.83.10.3316 3458185 PMC323504

[24] Engeland K. (2022) Cell cycle regulation: p53-p21-RB signaling. Cell Death Differ. 29, 946–960 10.1038/s41418-022-00988-z 35361964 PMC9090780

[25] Hernández Borrero L.J. and El-Deiry W.S. (2021) Tumor suppressor p53: Biology, signaling pathways, and therapeutic targeting. Biochim. Biophys. Acta Rev. Cancer 1876, 188556 10.1016/j.bbcan.2021.188556 33932560 PMC8730328

[26] Kastenhuber E.R. and Lowe S.W. (2017) Putting p53 in Context. Cell 170, 1062–1078 10.1016/j.cell.2017.08.028 28886379 PMC5743327

[27] Shamloo B. and Usluer S. (2019) p21 in Cancer Research. Cancers (Basel) 11, 1178 10.3390/cancers11081178 31416295 PMC6721478

[28] Xiong Y., Hannon G.J., Zhang H., Casso D., Kobayashi R. and Beach D. (1993) p21 is a universal inhibitor of cyclin kinases. Nature 366, 701–704 10.1038/366701a0 8259214

[29] Harper J.W., Adami G.R., Wei N., Keyomarsi K. and Elledge S.J. (1993) The p21 Cdk-interacting protein Cip1 is a potent inhibitor of G1 cyclin-dependent kinases. Cell 75, 805–816 10.1016/0092-8674(93)90499-G 8242751

[30] Yao Y., Gu X., Xu X., Ge S. and Jia R. (2022) Novel insights into RB1 mutation. Cancer Lett. 547, 215870 10.1016/j.canlet.2022.215870 35964818

[31] Rubin S.M., Sage J. and Skotheim J.M. (2020) Integrating Old and New Paradigms of G1/S Control. Mol. Cell 80, 183–192 10.1016/j.molcel.2020.08.020 32946743 PMC7582788

[32] Kent L.N. and Leone G. (2019) The broken cycle: E2F dysfunction in cancer. Nat. Rev. Cancer 19, 326–338 10.1038/s41568-019-0143-7 31053804

[33] Henley S.A. and Dick F.A. (2012) The retinoblastoma family of proteins and their regulatory functions in the mammalian cell division cycle. Cell Div. 7, 10 10.1186/1747-1028-7-10 22417103 PMC3325851

[34] Baus F., Gire V., Fisher D., Piette J. and Dulić V. (2003) Permanent cell cycle exit in G2 phase after DNA damage in normal human fibroblasts. EMBO J. 22, 3992–4002 10.1093/emboj/cdg387 12881433 PMC169060

[35] Leon K.E., Tangudu N.K., Aird K.M. and Buj R. (2021) Loss of p16: a bouncer of the immunological surveillance? Life (Basel) 11, 309 10.3390/life11040309 33918220 PMC8065641

[36] Baker D.J., Wijshake T., Tchkonia T., LeBrasseur N.K., Childs B.G., van de Sluis B. et al. (2011) Clearance of p16Ink4a-positive senescent cells delays ageing-associated disorders. Nature 479, 232–236 10.1038/nature10600 22048312 PMC3468323

[37] Baker D.J., Childs B.G., Durik M., Wijers M.E., Sieben C.J., Zhong J. et al. (2016) Naturally occurring p16(Ink4a)-positive cells shorten healthy lifespan. Nature 530, 184–189 10.1038/nature16932 26840489 PMC4845101

[38] Serra S. and Chetty R. (2018) p16. J. Clin. Pathol. 71, 853–858 10.1136/jclinpath-2018-205216 30076191

[39] Rayess H., Wang M.B. and Srivatsan E.S. (2012) Cellular senescence and tumor suppressor gene p16. Int. J. Cancer 130, 1715–1725 10.1002/ijc.27316 22025288 PMC3288293

[40] Alcorta D.A., Xiong Y., Phelps D., Hannon G., Beach D. and Barrett J.C. (1996) Involvement of the cyclin-dependent kinase inhibitor p16 (INK4a) in replicative senescence of normal human fibroblasts. Proc. Natl Acad. Sci. 93, 13742–13747 10.1073/pnas.93.24.13742 8943005 PMC19411

[41] Hara E., Smith R., Parry D., Tahara H., Stone S. and Peters G. (1996) Regulation of p16CDKN2 expression and its implications for cell immortalization and senescence. Mol. Cell. Biol. 16, 859–867 10.1128/MCB.16.3.859 8622687 PMC231066

[42] Noda A., Ning Y., Venable S.F., Pereira-Smith O.M. and Smith J.R. (1994) Cloning of senescent cell-derived inhibitors of DNA synthesis using an expression screen. Exp. Cell. Res. 211, 90–98 10.1006/excr.1994.1063 8125163

[43] Serrano M., Lin A.W., McCurrach M.E., Beach D. and Lowe S.W. (1997) Oncogenic ras provokes premature cell senescence associated with accumulation of p53 and p16INK4a. Cell 88, 593–602 10.1016/S0092-8674(00)81902-9 9054499

[44] Bernardes de Jesus B. and Blasco M.A. (2012) Assessing cell and organ senescence biomarkers. Circ. Res. 111, 97–109 10.1161/CIRCRESAHA.111.247866 22723221 PMC4824275

[45] Stein G.H., Drullinger L.F., Soulard A. and Dulić V. (1999) Differential roles for cyclin-dependent kinase inhibitors p21 and p16 in the mechanisms of senescence and differentiation in human fibroblasts. Mol. Cell. Biol. 19, 2109–2117 10.1128/MCB.19.3.2109 10022898 PMC84004

[46] Johmura Y., Shimada M., Misaki T., Naiki-Ito A., Miyoshi H., Motoyama N. et al. (2014) Necessary and sufficient role for a mitosis skip in senescence induction. Mol. Cell 55, 73–84 10.1016/j.molcel.2014.05.003 24910096

[47] Johmura Y. and Nakanishi M. (2016) Multiple facets of p53 in senescence induction and maintenance. Cancer Sci. 107, 1550–1555 10.1111/cas.13060 27560979 PMC5132285

[48] Coppé J.P., Patil C.K., Rodier F., Sun Y., Muñoz D.P., Goldstein J. et al. (2008) Senescence-associated secretory phenotypes reveal cell-nonautonomous functions of oncogenic RAS and the p53 tumor suppressor. PLoS Biol. 6, 2853–2868 10.1371/journal.pbio.0060301 19053174 PMC2592359

[49] Birch J. and Gil J. (2020) Senescence and the SASP: many therapeutic avenues. Genes Dev. 34, 1565–1576 10.1101/gad.343129.120 33262144 PMC7706700

[50] Wiley C.D., Liu S., Limbad C., Zawadzka A.M., Beck J., Demaria M. et al. (2019) SILAC analysis reveals increased secretion of hemostasis-related factors by senescent cells. Cell Rep. 28, 3329.e3325–3337.e3325 10.1016/j.celrep.2019.08.049 31553904 PMC6907691

[51] Basisty N., Kale A., Jeon O.H., Kuehnemann C., Payne T., Rao C. et al. (2020) A proteomic atlas of senescence-associated secretomes for aging biomarker development. PLoS Biol. 18, e3000599 10.1371/journal.pbio.3000599 31945054 PMC6964821

[52] Fumagalli M., Rossiello F., Clerici M., Barozzi S., Cittaro D., Kaplunov J.M. et al. (2012) Telomeric DNA damage is irreparable and causes persistent DNA-damage-response activation. Nat. Cell Biol. 14, 355–365 10.1038/ncb2466 22426077 PMC3717580

[53] Yousefzadeh M., Henpita C., Vyas R., Soto-Palma C., Robbins P. and Niedernhofer L. (2021) DNA damage-how and why we age? Elife 10, e62852 10.7554/eLife.62852 33512317 PMC7846274

[54] Ciccia A. and Elledge S.J. (2010) The DNA damage response: making it safe to play with knives. Mol. Cell 40, 179–204 10.1016/j.molcel.2010.09.019 20965415 PMC2988877

[55] Jackson S.P. and Bartek J. (2009) The DNA-damage response in human biology and disease. Nature 461, 1071–1078 10.1038/nature08467 19847258 PMC2906700

[56] Kumari R. and Jat P. (2021) Mechanisms of cellular senescence: cell cycle arrest and senescence associated secretory phenotype. Front Cell Dev. Biol. 9, 645593 10.3389/fcell.2021.645593 33855023 PMC8039141

[57] Petrova N.V., Velichko A.K., Razin S.V. and Kantidze O.L. (2016) Small molecule compounds that induce cellular senescence. Aging Cell 15, 999–1017 10.1111/acel.12518 27628712 PMC6398529

[58] Rossiello F., Jurk D., Passos J.F. and d'Adda di Fagagna F. (2022) Telomere dysfunction in ageing and age-related diseases. Nat. Cell Biol. 24, 135–147 10.1038/s41556-022-00842-x 35165420 PMC8985209

[59] Rothkamm K., Barnard S., Moquet J., Ellender M., Rana Z. and Burdak-Rothkamm S. (2015) DNA damage foci: meaning and significance. Environ. Mol. Mutagen. 56, 491–504 10.1002/em.21944 25773265

[60] Kinner A., Wu W., Staudt C. and Iliakis G. (2008) Gamma-H2AX in recognition and signaling of DNA double-strand breaks in the context of chromatin. Nucleic Acids Res. 36, 5678–5694 10.1093/nar/gkn550 18772227 PMC2553572

[61] Bonner W.M., Redon C.E., Dickey J.S., Nakamura A.J., Sedelnikova O.A., Solier S. et al. (2008) GammaH2AX and cancer. Nat. Rev. Cancer 8, 957–967 10.1038/nrc2523 19005492 PMC3094856

[62] Yang Y., Karsli-Uzunbas G., Poillet-Perez L., Sawant A., Hu Z.S., Zhao Y. et al. (2020) Autophagy promotes mammalian survival by suppressing oxidative stress and p53. Genes Dev. 34, 688–700 10.1101/gad.335570.119 32193353 PMC7197357

[63] Yousefzadeh M.J., Flores R.R., Zhu Y., Schmiechen Z.C., Brooks R.W., Trussoni C.E. et al. (2021) An aged immune system drives senescence and ageing of solid organs. Nature 594, 100–105 10.1038/s41586-021-03547-7 33981041 PMC8684299

[64] McHugh D. and Gil J. (2018) Senescence and aging: Causes, consequences, and therapeutic avenues. J. Cell Biol. 217, 65–77 10.1083/jcb.201708092 29114066 PMC5748990

[65] Mirman Z. and de Lange T. (2020) 53BP1: a DSB escort. Genes Dev. 34, 7–23 10.1101/gad.333237.119 31896689 PMC6938671

[66] Narita M., Nũnez S., Heard E., Narita M., Lin A.W., Hearn S.A. et al. (2003) Rb-mediated heterochromatin formation and silencing of E2F target genes during cellular senescence. Cell 113, 703–716 10.1016/S0092-8674(03)00401-X 12809602

[67] Howard B.H. (1996) Replicative senescence: considerations relating to the stability of heterochromatin domains. Exp. Gerontol. 31, 281–293 10.1016/0531-5565(95)00022-4 8706797

[68] Aird K.M. and Zhang R. (2013) Detection of senescence-associated heterochromatin foci (SAHF). Methods Mol. Biol. 965, 185–196 10.1007/978-1-62703-239-1_12 23296659 PMC3552318

[69] Zhang R., Chen W. and Adams P.D. (2007) Molecular dissection of formation of senescence-associated heterochromatin foci. Mol. Cell. Biol. 27, 2343–2358 10.1128/MCB.02019-06 17242207 PMC1820509

[70] Di Micco R., Sulli G., Dobreva M., Liontos M., Botrugno O.A., Gargiulo G. et al. (2011) Interplay between oncogene-induced DNA damage response and heterochromatin in senescence and cancer. Nat. Cell Biol. 13, 292–302 10.1038/ncb2170 21336312 PMC3918344

[71] Davalli P., Mitic T., Caporali A., Lauriola A. and D'Arca D.R.O.S. (2016) Cell senescence, and novel molecular mechanisms in aging and age-related diseases. Oxid. Med. Cell Longev. 2016, 3565127 10.1155/2016/3565127 27247702 PMC4877482

[72] Guo Y., Guan T., Shafiq K., Yu Q., Jiao X., Na D. et al. (2023) Mitochondrial dysfunction in aging. Ageing Res. Rev. 88, 101955 10.1016/j.arr.2023.101955 37196864

[73] Franci L., Tubita A., Bertolino F.M., Palma A., Cannino G., Settembre C. et al. (2022) MAPK15 protects from oxidative stress-dependent cellular senescence by inducing the mitophagic process. Aging Cell 21, e13620 10.1111/acel.13620 35642724 PMC9282834

[74] Zhong G., Qin S., Townsend D., Schulte B.A., Tew K.D. and Wang G.Y. (2019) Oxidative stress induces senescence in breast cancer stem cells. Biochem. Biophys. Res. Commun. 514, 1204–1209 10.1016/j.bbrc.2019.05.098 31109646 PMC6556123

[75] Chen Q.M., Prowse K.R., Tu V.C., Purdom S. and Linskens M.H. (2001) Uncoupling the senescent phenotype from telomere shortening in hydrogen peroxide-treated fibroblasts. Exp. Cell. Res. 265, 294–303 10.1006/excr.2001.5182 11302695

[76] Dumont P., Burton M., Chen Q.M., Gonos E.S., Frippiat C., Mazarati J.B. et al. (2000) Induction of replicative senescence biomarkers by sublethal oxidative stresses in normal human fibroblast. Free Radic. Biol. Med. 28, 361–373 10.1016/S0891-5849(99)00249-X 10699747

[77] Liao N., Shi Y., Zhang C., Zheng Y., Wang Y., Zhao B. et al. (2019) Antioxidants inhibit cell senescence and preserve stemness of adipose tissue-derived stem cells by reducing ROS generation during long-term in vitro expansion. Stem Cell Res. Ther. 10, 306 10.1186/s13287-019-1404-9 31623678 PMC6798439

[78] Yuan H., Xu Y., Luo Y., Zhang J.R., Zhu X.X. and Xiao J.H. (2022) Ganoderic acid D prevents oxidative stress-induced senescence by targeting 14-3-3ε to activate CaM/CaMKII/NRF2 signaling pathway in mesenchymal stem cells. Aging Cell 21, e13686 10.1111/acel.13686 35929187 PMC9470892

[79] Al-Dabet M.M., Shahzad K., Elwakiel A., Sulaj A., Kopf S., Bock F. et al. (2022) Reversal of the renal hyperglycemic memory in diabetic kidney disease by targeting sustained tubular p21 expression. Nat. Commun. 13, 5062 10.1038/s41467-022-32477-9 36030260 PMC9420151

[80] Zhu K., Kakehi T., Matsumoto M., Iwata K., Ibi M., Ohshima Y. et al. (2015) NADPH oxidase NOX1 is involved in activation of protein kinase C and premature senescence in early stage diabetic kidney. Free Radic. Biol. Med. 83, 21–30 10.1016/j.freeradbiomed.2015.02.009 25701431

[81] Kakoki M., Kizer C.M., Yi X., Takahashi N., Kim H.S., Bagnell C.R. et al. (2006) Senescence-associated phenotypes in Akita diabetic mice are enhanced by absence of bradykinin B2 receptors. J. Clin. Invest. 116, 1302–1309 10.1172/JCI26958 16604193 PMC1430357

[82] Ji B., Cheng B., Pan Y., Wang C., Chen J. and Bai B. (2017) Neuroprotection of bradykinin/bradykinin B2 receptor system in cerebral ischemia. Biomed. Pharmacother. 94, 1057–1063 10.1016/j.biopha.2017.08.042 28810528

[83] Fu C., Cao Y., Li B., Xu R., Sun Y. and Yao Y. (2019) Bradykinin protects cardiac c-kit positive cells from high-glucose-induced senescence through B2 receptor signaling pathway. J. Cell. Biochem. 120, 17731–17743 10.1002/jcb.29039 31119778

[84] Fu C., Li B., Sun Y., Ma G. and Yao Y. (2015) Bradykinin inhibits oxidative stress-induced senescence of endothelial progenitor cells through the B2R/AKT/RB and B2R/EGFR/RB signal pathways. Oncotarget 6, 24675–24689 10.18632/oncotarget.5071 26360782 PMC4694787

[85] Vashistha H., Marrero L., Reiss K., Cohen A.J., Malhotra A., Javed T. et al. (2018) Aging phenotype(s) in kidneys of diabetic mice are p66ShcA dependent. Am. J. Physiol. Renal. Physiol. 315, F1833–F1842 10.1152/ajprenal.00608.2017 30207172 PMC6336986

[86] Di Stefano V., Cencioni C., Zaccagnini G., Magenta A., Capogrossi M.C. and Martelli F. (2009) p66ShcA modulates oxidative stress and survival of endothelial progenitor cells in response to high glucose. Cardiovasc. Res. 82, 421–429 10.1093/cvr/cvp082 19261622

[87] Menini S., Amadio L., Oddi G., Ricci C., Pesce C., Pugliese F. et al. (2006) Deletion of p66Shc longevity gene protects against experimental diabetic glomerulopathy by preventing diabetes-induced oxidative stress. Diabetes 55, 1642–1650 10.2337/db05-1477 16731826

[88] Jha J.C., Gray S.P., Barit D., Okabe J., El-Osta A., Namikoshi T. et al. (2014) Genetic targeting or pharmacologic inhibition of NADPH oxidase nox4 provides renoprotection in long-term diabetic nephropathy. J. Am. Soc. Nephrol.:JASN 25, 1237–1254 10.1681/ASN.2013070810 24511132 PMC4033375

[89] Jha J.C., Banal C., Okabe J., Gray S.P., Hettige T., Chow B.S.M. et al. (2017) NADPH oxidase Nox5 accelerates renal injury in diabetic nephropathy. Diabetes 66, 2691–2703 10.2337/db16-1585 28747378

[90] Verzola D., Gandolfo M.T., Gaetani G., Ferraris A., Mangerini R., Ferrario F. et al. (2008) Accelerated senescence in the kidneys of patients with type 2 diabetic nephropathy. Am. J. Physiol. Renal. Physiol. 295, F1563–F1573 10.1152/ajprenal.90302.2008 18768588

[91] Chen K., Dai H., Yuan J., Chen J., Lin L., Zhang W. et al. (2018) Optineurin-mediated mitophagy protects renal tubular epithelial cells against accelerated senescence in diabetic nephropathy. Cell Death Dis 9, 105 10.1038/s41419-017-0127-z 29367621 PMC5833650

[92] Zhang X., Chen X., Wu D., Liu W., Wang J., Feng Z. et al. (2006) Downregulation of connexin 43 expression by high glucose induces senescence in glomerular mesangial cells. J. Am. Soc. Nephrol. 17, 1532–1542 10.1681/ASN.2005070776 16675599

[93] Fang Y., Chen B., Gong A.Y., Malhotra D.K., Gupta R., Dworkin L.D. et al. (2021) The ketone body β-hydroxybutyrate mitigates the senescence response of glomerular podocytes to diabetic insults. Kidney Int. 100, 1037–1053 10.1016/j.kint.2021.06.031 34246657 PMC8889914

[94] Yu S., Cheng Y., Li B., Xue J., Yin Y., Gao J. et al. (2020) M1 macrophages accelerate renal glomerular endothelial cell senescence through reactive oxygen species accumulation in streptozotocin-induced diabetic mice. Int. Immunopharmacol. 81, 106294 10.1016/j.intimp.2020.106294 32062081

[95] Bian X., Griffin T.P., Zhu X., Islam M.N., Conley S.M., Eirin A. et al. (2019) Senescence marker activin A is increased in human diabetic kidney disease: association with kidney function and potential implications for therapy. BMJ Open Diab. Res. Care 7, e000720 10.1136/bmjdrc-2019-000720 PMC6936543 31908790

[96] Sangoi M.B., de Carvalho J.A., Tatsch E., Hausen B.S., Bollick Y.S., Londero S.W. et al. (2016) Urinary inflammatory cytokines as indicators of kidney damage in type 2 diabetic patients. Clin. Chim. Acta 460, 178–183 10.1016/j.cca.2016.06.028 27353644

[97] Yu C. and Xiao J.H. (2021) The Keap1-Nrf2 system: a mediator between oxidative stress and aging. Oxid. Med. Cell Longev. 2021, 6635460 10.1155/2021/6635460 34012501 PMC8106771

[98] Wang Z., Han N., Zhao K., Li Y., Chi Y. and Wang B. (2019) Protective effects of pyrroloquinoline quinine against oxidative stress-induced cellular senescence and inflammation in human renal tubular epithelial cells via Keap1/Nrf2 signaling pathway. Int. Immunopharmacol. 72, 445–453 10.1016/j.intimp.2019.04.040 31035086

[99] Wang L., Chen P. and Xiao W. (2021) β-Hydroxybutyrate as an anti-aging metabolite. Nutrients 13, 3420 10.3390/nu13103420 34684426 PMC8540704

[100] Chen M., Fang Y., Ge Y., Qiu S., Dworkin L. and Gong R. (2024) The redox-sensitive GSK3β is a key regulator of glomerular podocyte injury in type 2 diabetic kidney disease. Redox Biol. 72, 103127 10.1016/j.redox.2024.103127 38527400 PMC10979123

[101] Kang C. and Elledge S.J. (2016) How autophagy both activates and inhibits cellular senescence. Autophagy 12, 898–899 10.1080/15548627.2015.1121361 27129029 PMC4854549

[102] Kaushik S., Tasset I., Arias E., Pampliega O., Wong E., Martinez-Vicente M. et al. (2021) Autophagy and the hallmarks of aging. Ageing Res. Rev. 72, 101468 10.1016/j.arr.2021.101468 34563704 PMC8616816

[103] Shi M., Yang S., Zhu X., Sun D., Sun D., Jiang X. et al. (2019) The RAGE/STAT5/autophagy axis regulates senescence in mesangial cells. Cell. Signal. 62, 109334 10.1016/j.cellsig.2019.05.019 31158467

[104] Chen L., Mei G., Jiang C., Cheng X., Li D., Zhao Y. et al. (2021) Carbon monoxide alleviates senescence in diabetic nephropathy by improving autophagy. Cell Prolif. 54, e13052 10.1111/cpr.13052 33963627 PMC8168421

[105] Luo Y., Zhang L., Su N., Liu L. and Zhao T. (2024) YME1L-mediated mitophagy protects renal tubular cells against cellular senescence under diabetic conditions. Biol. Res. 57, 10 10.1186/s40659-024-00487-0 38494498 PMC10946153

[106] Kang C., Xu Q., Martin T.D., Li M.Z., Demaria M., Aron L. et al. (2015) The DNA damage response induces inflammation and senescence by inhibiting autophagy of GATA4. Science (New York, N.Y.) 349, aaa5612 10.1126/science.aaa5612 26404840 PMC4942138

[107] Chen K., Chen J., Wang L., Yang J., Xiao F., Wang X. et al. (2020) Parkin ubiquitinates GATA4 and attenuates the GATA4/GAS1 signaling and detrimental effects on diabetic nephropathy. FASEB J. 34, 8858–8875 10.1096/fj.202000053R 32436607

[108] Sankrityayan H., Oza M.J., Kulkarni Y.A., Mulay S.R. and Gaikwad A.B. (2019) ER stress response mediates diabetic microvascular complications. Drug Discov. Today 24, 2247–2257 10.1016/j.drudis.2019.08.003 31430543

[109] Ni L., Yuan C. and Wu X. (2021) Endoplasmic reticulum stress in diabetic nephrology: regulation, pathological role, and therapeutic potential. Oxid. Med. Cell Longev. 2021, 7277966 10.1155/2021/7277966 34394833 PMC8355967

[110] Liu J., Yang J.R., Chen X.M., Cai G.Y., Lin L.R. and He Y.N. (2015) Impact of ER stress-regulated ATF4/p16 signaling on the premature senescence of renal tubular epithelial cells in diabetic nephropathy. Am. J. Physiol. Cell Physiol. 308, C621–C630 10.1152/ajpcell.00096.2014 25567807

[111] Liu J., Huang K., Cai G.Y., Chen X.M., Yang J.R., Lin L.R. et al. (2014) Receptor for advanced glycation end-products promotes premature senescence of proximal tubular epithelial cells via activation of endoplasmic reticulum stress-dependent p21 signaling. Cell. Signal. 26, 110–121 10.1016/j.cellsig.2013.10.002 24113348

[112] Tu B.P. and Weissman J.S. (2004) Oxidative protein folding in eukaryotes: mechanisms and consequences. J. Cell Biol. 164, 341–346 10.1083/jcb.200311055 14757749 PMC2172237

[113] Zhang Z., Zhang L., Zhou L., Lei Y., Zhang Y. and Huang C. (2019) Redox signaling and unfolded protein response coordinate cell fate decisions under ER stress. Redox Biol. 25, 101047 10.1016/j.redox.2018.11.005 30470534 PMC6859529

[114] Katakura Y., Nakata E., Miura T. and Shirahata S. (1999) Transforming growth factor beta triggers two independent-senescence programs in cancer cells. Biochem. Biophys. Res. Commun. 255, 110–115 10.1006/bbrc.1999.0129 10082664

[115] Reynisdóttir I., Polyak K., Iavarone A. and Massagué J. (1995) Kip/Cip and Ink4 Cdk inhibitors cooperate to induce cell cycle arrest in response to TGF-beta. Genes Dev. 9, 1831–1845 10.1101/gad.9.15.1831 7649471

[116] Datto M.B., Li Y., Panus J.F., Howe D.J., Xiong Y. and Wang X.F. (1995) Transforming growth factor beta induces the cyclin-dependent kinase inhibitor p21 through a p53-independent mechanism. Proc. Natl Acad. Sci. 92, 5545–5549 10.1073/pnas.92.12.5545 7777546 PMC41732

[117] Tominaga K. and Suzuki H.I. (2019) TGF-β signaling in cellular senescence and aging-related pathology. Int. J. Mol. Sci. 20, 5002 10.3390/ijms20205002 31658594 PMC6834140

[118] Yang X.H., Zhang B.L., Zhang X.M., Tong J.D., Gu Y.H., Guo L.L. et al. (2020) EGCG Attenuates renal damage via reversing klotho hypermethylation in diabetic db/db mice and HK-2 cells. Oxid. Med. Cell Longev. 2020, 6092715 10.1155/2020/6092715 32908633 PMC7474393

[119] Eleftheriadis T., Pissas G., Filippidis G., Efthymiadi M., Liakopoulos V. and Stefanidis I. (2022) Dapagliflozin prevents high-glucose-induced cellular senescence in renal tubular epithelial cells. Int. J. Mol. Sci. 23, 16107 10.3390/ijms232416107 36555751 PMC9781434

[120] Ziyadeh F.N., Hoffman B.B., Han D.C., Iglesias-De La Cruz M.C., Hong S.W., Isono M. et al. (2000) Long-term prevention of renal insufficiency, excess matrix gene expression, and glomerular mesangial matrix expansion by treatment with monoclonal antitransforming growth factor-beta antibody in db/db diabetic mice. Proc. Natl Acad. Sci. 97, 8015–8020 10.1073/pnas.120055097 10859350 PMC16662

[121] Houlihan C.A., Akdeniz A., Tsalamandris C., Cooper M.E., Jerums G. and Gilbert R.E. (2002) Urinary transforming growth factor-beta excretion in patients with hypertension, type 2 diabetes, and elevated albumin excretion rate: effects of angiotensin receptor blockade and sodium restriction. Diabetes Care. 25, 1072–1077 10.2337/diacare.25.6.1072 12032117

[122] Petersen M., Thorikay M., Deckers M., van Dinther M., Grygielko E.T., Gellibert F. et al. (2008) Oral administration of GW788388, an inhibitor of TGF-beta type I and II receptor kinases, decreases renal fibrosis. Kidney Int. 73, 705–715 10.1038/sj.ki.5002717 18075500

[123] Huynh P. and Chai Z. (2019) Transforming growth factor β (TGFβ) and related molecules in chronic kidney disease (CKD). Clin. Sci. (Lond.) 133, 287–313 10.1042/CS20180438 30683713

[124] Pham Y., Tu Y., Wu T., Allen T.J., Calkin A.C., Watson A.M. et al. (2010) Cell division autoantigen 1 plays a profibrotic role by modulating downstream signalling of TGF-beta in a murine diabetic model of atherosclerosis. Diabetologia 53, 170–179 10.1007/s00125-009-1555-9 19847393

[125] Toh B.H., Tu Y., Cao Z., Cooper M.E. and Chai Z. (2010) Role of cell division autoantigen 1 (CDA1) in cell proliferation and fibrosis. Genes (Basel) 1, 335–348 10.3390/genes1030335 24710090 PMC3966230

[126] Chai Z., Sarcevic B., Mawson A. and Toh B.H. (2001) SET-related cell division autoantigen-1 (CDA1) arrests cell growth. J. Biol. Chem. 276, 33665–33674 10.1074/jbc.M007681200 11395479

[127] Tu Y., Wu W., Wu T., Cao Z., Wilkins R., Toh B.H. et al. (2007) Antiproliferative autoantigen CDA1 transcriptionally up-regulates p21(Waf1/Cip1) by activating p53 and MEK/ERK1/2 MAPK pathways. J. Biol. Chem. 282, 11722–11731 10.1074/jbc.M609623200 17317670

[128] Magni M., Buscemi G., Maita L., Peng L., Chan S.Y., Montecucco A. et al. (2019) TSPYL2 is a novel regulator of SIRT1 and p300 activity in response to DNA damage. Cell Death Differ. 26, 918–931 10.1038/s41418-018-0168-6 30050056 PMC6461906

[129] Chai Z., Wu T., Dai A., Huynh P., Koentgen F., Krippner G. et al. (2019) Targeting the CDA1/CDA1BP1 axis retards renal fibrosis in experimental diabetic nephropathy. Diabetes 68, 395–408 10.2337/db18-0712 30425061

[130] Tu Y., Wu T., Dai A., Pham Y., Chew P., de Haan J.B. et al. (2011) Cell division autoantigen 1 enhances signaling and the profibrotic effects of transforming growth factor-β in diabetic nephropathy. Kidney Int. 79, 199–209 10.1038/ki.2010.374 20962744

[131] Chai Z., Dai A., Tu Y., Li J., Wu T., Wang Y. et al. (2013) Genetic deletion of cell division autoantigen 1 retards diabetes-associated renal injury. J. Am. Soc. Nephrol. 24, 1782–1792 10.1681/ASN.2013010060 23929772 PMC3810085

[132] Coughlan M.T., Ziemann M., Laskowski A., Woodruff T.M. and Tan S.M. (2022) Valproic acid attenuates cellular senescence in diabetic kidney disease through the inhibition of complement C5a receptors. Sci. Rep. 12, 20278 10.1038/s41598-022-24851-w 36434087 PMC9700697

[133] Tang S.C.W. and Yiu W.H. (2020) Innate immunity in diabetic kidney disease. Nat. Rev. Nephrol. 16, 206–222 31942046 10.1038/s41581-019-0234-4

[134] Meng X.M., Nikolic-Paterson D.J. and Lan H.Y. (2016) TGF-β: the master regulator of fibrosis. Nat. Rev. Nephrol. 12, 325–338 27108839 10.1038/nrneph.2016.48

[135] Braun H., Schmidt B.M., Raiss M., Baisantry A., Mircea-Constantin D., Wang S. et al. (2012) Cellular senescence limits regenerative capacity and allograft survival. J. Am. Soc. Nephrol. 23, 1467–1473 10.1681/ASN.2011100967 22797186 PMC3431409

[136] Luo C., Zhou S., Zhou Z., Liu Y., Yang L., Liu J. et al. (2018) Wnt9a promotes renal fibrosis by accelerating cellular senescence in tubular epithelial cells. J. Am. Soc. Nephrol. 29, 1238–1256 10.1681/ASN.2017050574 29440280 PMC5875944

[137] Jia C., Ke-Hong C., Fei X., Huan-Zi D., Jie Y., Li-Ming W. et al. (2020) Decoy receptor 2 mediation of the senescent phenotype of tubular cells by interacting with peroxiredoxin 1 presents a novel mechanism of renal fibrosis in diabetic nephropathy. Kidney Int. 98, 645–662 10.1016/j.kint.2020.03.026 32739204

[138] Bonventre J.V. (2014) Maladaptive proximal tubule repair: cell cycle arrest. Nephron Clin. Pract. 127, 61–64 10.1159/000363673 25343823

[139] Kitamura S., Yamasaki Y., Kinomura M., Sugaya T., Sugiyama H., Maeshima Y. et al. (2005) Establishment and characterization of renal progenitor like cells from S3 segment of nephron in rat adult kidney. FASEB J. 19, 1789–1797 10.1096/fj.05-3942com 16260649

[140] Lazzeri E., Crescioli C., Ronconi E., Mazzinghi B., Sagrinati C., Netti G.S. et al. (2007) Regenerative potential of embryonic renal multipotent progenitors in acute renal failure. J. Am. Soc. Nephrol. 18, 3128–3138 10.1681/ASN.2007020210 17978305

[141] Romagnani P. and Remuzzi G. (2014) CD133+ renal stem cells always co-express CD24 in adult human kidney tissue. Stem Cell Res. 12, 828–829 10.1016/j.scr.2013.12.011 24467938

[142] Zou X., Kwon S.H., Jiang K., Ferguson C.M., Puranik A.S., Zhu X. et al. (2018) Renal scattered tubular-like cells confer protective effects in the stenotic murine kidney mediated by release of extracellular vesicles. Sci. Rep. 8, 1263 10.1038/s41598-018-19750-y 29352176 PMC5775303

[143] Chen X.J., Kim S.R., Jiang K., Ferguson C.M., Tang H., Zhu X.Y. et al. (2021) Renovascular disease induces senescence in renal scattered tubular-like cells and impairs their reparative potency. Hypertension (Dallas, Tex.: 1979) 77, 507–518 10.1161/HYPERTENSIONAHA.120.16218 33390051 PMC7808550

[144] Basu A. (2022) The interplay between apoptosis and cellular senescence: Bcl-2 family proteins as targets for cancer therapy. Pharmacol. Ther. 230, 107943 10.1016/j.pharmthera.2021.107943 34182005

[145] Pignolo R.J., Passos J.F., Khosla S., Tchkonia T. and Kirkland J.L. (2020) Reducing senescent cell burden in aging and disease. Trends Mol. Med. 26, 630–638 10.1016/j.molmed.2020.03.005 32589933 PMC7857028

[146] Deryabin P.I., Shatrova A.N. and Borodkina A.V. (2021) Apoptosis resistance of senescent cells is an intrinsic barrier for senolysis induced by cardiac glycosides. Cell. Mol. Life Sci. 78, 7757–7776 10.1007/s00018-021-03980-x 34714358 PMC8629786

[147] Chen J., Chen K.H., Wang L.M., Luo J., Zheng Q.Y. and He Y.N. (2022) Decoy receptor 2 mediates the apoptosis-resistant phenotype of senescent renal tubular cells and accelerates renal fibrosis in diabetic nephropathy. Cell Death Dis. 13, 522 10.1038/s41419-022-04972-w 35661704 PMC9166763

[148] Baar M.P., Brandt R.M.C., Putavet D.A., Klein J.D.D., Derks K.W.J., Bourgeois B.R.M. et al. (2017) Targeted apoptosis of senescent cells restores tissue homeostasis in response to chemotoxicity and aging. Cell 169, 132.e116–147.e116 10.1016/j.cell.2017.02.031 28340339 PMC5556182

[149] Ren H., Shao Y., Wu C., Ma X., Lv C. and Wang Q. (2020) Metformin alleviates oxidative stress and enhances autophagy in diabetic kidney disease via AMPK/SIRT1-FoxO1 pathway. Mol. Cell. Endocrinol. 500, 110628 10.1016/j.mce.2019.110628 31647955

[150] Kawanami D., Takashi Y. and Tanabe M. (2020) Significance of metformin use in diabetic kidney disease. Int. J. Mol. Sci. 21, 4239 10.3390/ijms21124239 32545901 PMC7352798

[151] Jiang X., Ruan X.L., Xue Y.X., Yang S., Shi M. and Wang L.N. (2020) Metformin reduces the senescence of renal tubular epithelial cells in diabetic nephropathy via the MBNL1/miR-130a-3p/STAT3 pathway. Oxid. Med. Cell Longev. 2020, 8708236 10.1155/2020/8708236 32104542 PMC7035567

[152] Liang D., Li Z., Feng Z., Yuan Z., Dai Y., Wu X. et al. (2022) Metformin improves the senescence of renal tubular epithelial cells in a high-glucose state through E2F1. Front Pharmacol. 13, 926211 10.3389/fphar.2022.926211 35814218 PMC9262145

[153] Khan J., Pernicova I., Nisar K. and Korbonits M. (2023) Mechanisms of ageing: growth hormone, dietary restriction, and metformin. Lancet Diab. Endocrinol. 11, 261–281 10.1016/S2213-8587(23)00001-3 36848915

[154] Noren Hooten N., Martin-Montalvo A., Dluzen D.F., Zhang Y., Bernier M., Zonderman A.B. et al. (2016) Metformin-mediated increase in DICER1 regulates microRNA expression and cellular senescence. Aging Cell 15, 572–581 10.1111/acel.12469 26990999 PMC4854919

[155] Śmieszek A., Stręk Z., Kornicka K., Grzesiak J., Weiss C. and Marycz K. (2017) Antioxidant and anti-senescence effect of metformin on mouse olfactory ensheathing cells (mOECs) may be associated with increased brain-derived neurotrophic factor levels-an ex vivo study. Int. J. Mol. Sci. 18, 872 10.3390/ijms18040872 28425952 PMC5412453

[156] Fang J., Yang J., Wu X., Zhang G., Li T., Wang X. et al. (2018) Metformin alleviates human cellular aging by upregulating the endoplasmic reticulum glutathione peroxidase 7. Aging Cell 17, e12765 10.1111/acel.12765 29659168 PMC6052468

[157] Chen D., Xia D., Pan Z., Xu D., Zhou Y., Wu Y. et al. (2016) Metformin protects against apoptosis and senescence in nucleus pulposus cells and ameliorates disc degeneration in vivo. Cell Death Dis 7, e2441 10.1038/cddis.2016.334 27787519 PMC5133996

[158] Kim M.N., Moon J.H. and Cho Y.M. (2021) Sodium-glucose cotransporter-2 inhibition reduces cellular senescence in the diabetic kidney by promoting ketone body-induced NRF2 activation. Diabetes Obes. Metab. 23, 2561–2571 10.1111/dom.14503 34318973

[159] Li J., Albajrami O., Zhuo M., Hawley C.E. and Paik J.M. (2020) Decision algorithm for prescribing SGLT2 inhibitors and GLP-1 receptor agonists for diabetic kidney disease. Clin. J. Am. Soc. Nephrol. 15, 1678–1688 10.2215/CJN.02690320 32518100 PMC7646234

[160] Nian S., Mi Y., Ren K., Wang S., Li M. and Yang D. (2022) The inhibitory effects of Dulaglutide on cellular senescence against high glucose in human retinal endothelial cells. Hum. Cell 35, 995–1004 10.1007/s13577-022-00703-7 35583801

[161] Kawanami D. and Takashi Y. (2020) GLP-1 receptor agonists in diabetic kidney disease: from clinical outcomes to mechanisms. Front Pharmacol. 11, 967 10.3389/fphar.2020.00967 32694999 PMC7338581

[162] Rosenstock J., Perkovic V., Johansen O.E., Cooper M.E., Kahn S.E., Marx N. et al. (2019) Effect of linagliptin vs placebo on major cardiovascular events in adults with type 2 diabetes and high cardiovascular and renal risk: the CARMELINA randomized clinical trial. JAMA 321, 69–79 10.1001/jama.2018.18269 30418475 PMC6583576

[163] Mosenzon O., Leibowitz G., Bhatt D.L., Cahn A., Hirshberg B., Wei C. et al. (2017) Effect of saxagliptin on renal outcomes in the SAVOR-TIMI 53 Trial. Diabetes Care 40, 69–76 10.2337/dc16-0621 27797925

[164] Chalmoukou K., Polyzos D., Manta E., Tatakis F., Konstantinidis D., Thomopoulos C. et al. (2022) Renal outcomes associated with glucose-lowering agents: Systematic review and meta-analysis of randomized outcome trials. Eur. J. Intern. Med. 97, 78–85 10.1016/j.ejim.2021.12.018 34953655

[165] Meng Q., Chen C., Yang N., Gololobova O., Shi C., Dunn C.A. et al. (2023) Surfaceome analysis of extracellular vesicles from senescent cells uncovers uptake repressor DPP4. Proc. Natl Acad. Sci. 120, e2219801120 10.1073/pnas.2219801120 37862381 PMC10614838

[166] Childs B.G., Gluscevic M., Baker D.J., Laberge R.M., Marquess D., Dananberg J. et al. (2017) Senescent cells: an emerging target for diseases of ageing. Nat. Rev. Drug Discovery 16, 718–735 10.1038/nrd.2017.116 28729727 PMC5942225

[167] Sweeney M., Cook S.A. and Gil J. (2023) Therapeutic opportunities for senolysis in cardiovascular disease. FEBS J. 290, 1235–1255 10.1111/febs.16351 35015342 PMC10952275

[168] Brattås M.K., Reikvam H. and Tvedt T.H.A. (2019) Bruserud, Ø. Dasatinib as an investigational drug for the treatment of Philadelphia chromosome-positive acute lymphoblastic leukemia in adults. Expert Opin. Investig. Drugs 28, 411–420 10.1080/13543784.2019.1597052 30916583

[169] Shen P., Lin W., Deng X., Ba X., Han L., Chen Z. et al. (2021) Potential implications of quercetin in autoimmune diseases. Front Immunol. 12, 689044 10.3389/fimmu.2021.689044 34248976 PMC8260830

[170] Zhu Y., Tchkonia T., Pirtskhalava T., Gower A.C., Ding H., Giorgadze N. et al. (2015) The Achilles' heel of senescent cells: from transcriptome to senolytic drugs. Aging Cell 14, 644–658 10.1111/acel.12344 25754370 PMC4531078

[171] Hickson L.J., Langhi Prata L.G.P., Bobart S.A., Evans T.K., Giorgadze N., Hashmi S.K. et al. (2019) Senolytics decrease senescent cells in humans: Preliminary report from a clinical trial of Dasatinib plus Quercetin in individuals with diabetic kidney disease. EBioMedicine 47, 446–456 10.1016/j.ebiom.2019.08.069 31542391 PMC6796530

[172] Tesch G.H., Ma F.Y., Ozols E. and Nikolic-Paterson D.J. (2024) Intervention treatment reducing cellular senescence inhibits tubulointerstitial fibrosis in diabetic mice following acute kidney injury. Clin. Sci. (Lond.) 138, 309–326 10.1042/CS20231698 38391050 PMC10914710

[173] Lucas V., Cavadas C. and Aveleira C.A. (2023) Cellular senescence: from mechanisms to current biomarkers and senotherapies. Pharmacol. Rev. 75, 675–713 10.1124/pharmrev.122.000622 36732079

[174] Shin J.H., Jeon H.J., Park J. and Chang M.S. (2016) Epigallocatechin-3-gallate prevents oxidative stress-induced cellular senescence in human mesenchymal stem cells via Nrf2. Int. J. Mol. Med. 38, 1075–1082 10.3892/ijmm.2016.2694 27498709 PMC5029951

[175] Sun W., Liu X., Zhang H., Song Y., Li T., Liu X. et al. (2017) Epigallocatechin gallate upregulates NRF2 to prevent diabetic nephropathy via disabling KEAP1. Free Radic. Biol. Med. 108, 840–857 10.1016/j.freeradbiomed.2017.04.365 28457936

